# Validity and predictability of mid-upper arm circumference for nutrition screening in outpatient preschoolers with cerebral palsy

**DOI:** 10.3389/fnut.2025.1609032

**Published:** 2025-09-05

**Authors:** Hongyu Zhou, Tingting Peng, Mou Wei, Jingbo Zhang, Yiting Zhao, Wen Le, Danxia Fan, Shaihong Qiu, Yuai Zheng, Qiujin Lin, Yun Zheng, Liying Ma, Jing Zhang, Jinling Li, Jinhua Lu, Hongmei Tang, Lu He, Kaishou Xu

**Affiliations:** ^1^Department of Rehabilitation, Guangzhou Women and Children Medical Center, Guangzhou Medical University, Guangzhou, China; ^2^Department of Neonatology, Women and Children's Medical Center, Guangzhou Medical University, Guangzhou, China; ^3^Department of Sport Rehabilitation, Shanghai University of Sport, Shanghai, China; ^4^School of Nursing, Guangdong Pharmaceutical University, Guangzhou, China; ^5^Division of Birth Cohort Study, Guangzhou Women and Children’s Medical Center, Guangzhou Medical University, Guangzhou, China

**Keywords:** cerebral palsy, malnutrition, undernutrition, overnutrition, mid-upper arm circumference, cutoffs, preschoolers

## Abstract

**Aim:**

Evaluating the nutritional status of children with cerebral palsy (CP) is difficult due to spasticity and contractures. Mid-upper arm circumference (MUAC) is a potential screening tool for malnutrition in children with CP, but its effectiveness is unproven. This study aims to provide evidence on the psychometric qualities of MUAC for clinical use and establish optimal cutoffs for preschoolers with CP.

**Methods:**

Children with CP aged 12–60 months (*n* = 937) were recruited from 24 hospitals across 13 provinces in China for the cross-sectional study, while those had genetic or metabolic diseases were excluded. Weight, length/height and MUAC were obtained from participants. Weight and length/height were calculated into Z scores by using WHO Anthro software to assess the nutritional status. The sensitivity and specificity of the WHO-recommended MUAC cutoffs were calculated. The Spearman’s rank correlation, Receiver operating characteristic (ROC) curve, and Youden Index were conducted to establish the optimal MUAC cutoffs for preschoolers with CP.

**Results:**

Compared to Z score cutoffs, WHO-recommended MUAC cutoffs showed high specificity but low sensitivity for malnutrition. MUAC significantly correlated with weight-for-length/height Z score (*r* = 0.606), weight-for-age Z score (*r* = 0.557), length/height-for-age Z score (*r* = 0.276), and BMI-for-age Z score (*r* = 0.575). The optimal MUAC cutoffs for mild, moderate, and severe undernutrition were 15.35, 15.05, and 14.35 cm, respectively; the optimal cutoffs for overweight and obesity were 17.55 and 20.4 cm, respectively.

**Conclusion:**

Our study suggests that MUAC is a useful tool for screening the nutritional status of children with CP. However, the WHO-recommended MUAC cut-off may not be suitable for preschool with CP. We estimated that the optimal MUAC cutoffs were 15.35 cm for mild undernutrition, 15.05 cm for moderate undernutrition, and 14.35 cm for severe undernutrition, and 17.55 cm for overweight and 20.4 cm for obesity in preschool with CP.

**Clinical trial registration:**

www.chictr.org.cn, ChiCTR2000033869.

## Introduction

Cerebral palsy (CP), with a prevalence of 2–3.5‰, affects about 50 million people worldwide (1, 2). Malnutrition has been recognized as a common and detrimental condition associated with CP. 57.6–80% of children with CP suffer from malnutrition for various reasons, such as feeding and swallowing difficulties, abnormal muscle tone, and gastrointestinal problems (3–6). Malnutrition affects children’s overall health, growth, and participation in educational and social activities (7). There is a growing emphasis on nutrition screening and assessment as they facilitate early recognition and management of malnutrition in CP children (8).

Anthropometric measurements are widely used to assess patients’ nutritional status and health. However, it is difficult to collect core anthropometric data, especially weight and height, from children with CP in a busy clinic due to their compromised motor ability and abnormal position (9). Mid-upper arm circumference (MUAC) is an independent and easily obtained measure of the upper arm’s subcutaneous fat, muscle, and bone. Recent studies have shown that MUAC is a reliable tool for indirectly assessing growth and changes in caloric and protein intake in children, pregnant women, and the elderly (10–12). The World Health Organization (WHO) recommends MUAC as a diagnostic tool for severe acute malnutrition in typically developing children. However, the current MUAC cutoffs proposed by WHO only identify those in moderate (between 11.5 and 12.5 cm) or severe (less than 11.5 cm) undernutrition (13, 14). Although some studies have used MUAC as a proxy to assess the nutritional status and other clinical characteristics of children with CP, few have used it for nutrition screening (15–17). Furthermore, to our knowledge, specific MUAC cutoffs to screen for malnutrition in children with CP have not been reported (18).

Recently, MUAC was found to be currently detecting overweight children though the cutoffs in different countries were established inconsistently (19). MUAC, combined with age and the Gross Motor Function Classification System (GMFCS) level, can estimate body weight in children with CP (20). As children with CP were reported to present altered body composition (21, 22), which may affect their nutritional status, it is crucial to establish the optimal MUAC cutoffs for nutrition screening.

Therefore, this study aims to identify the optimal MUAC cutoff values for predicting malnutrition (undernutrition and overnutrition) in outpatient preschoolers with CP aged 12–60 months.

## Materials and methods

### Study design and participants

This study is part of a multicenter cross-sectional study (ChiCTR2000033869) which was conducted to investigate the nutritional characteristics of children with CP in China (6). The enrolled participants were from 24 hospitals across 13 provinces in China. Children aged 12–60 months with a diagnosis of CP and not offered standardized nutritional intervention previously were included in this study at their first outpatient visits from July 2020 to December 2021. Those who had genetic or metabolic diseases were excluded. And the GMFCS level, the Eating and Drinking Ability Classification System (EDACS) or Mini-EDACS level were determined by experienced clinicians who received standard training.

This study was approved by the Ethics Committee of Guangzhou Women and Children’s Medical Center (GWCMC) (approval number: 2020–29602) and registered at Chinese Clinical Trial Registry (registration number: ChiCTR2000033869).

### Data collection

#### GMFCS level

The GMFCS is a system that classifies gross motor function into five levels for people with CP (23, 24). The criteria for each level are based on age and were created with the expectation that children would remain at the same GMFCS level throughout their childhood and adolescence. Level I: Walks without limitations but experiences limitations in performing complex and skillful motor activities. Level II: Can walk without assistive devices but has limitations in outdoor and community settings. Level III: Requires assistive devices for walking and has limitations in walking outdoors and in the community. Level IV: Cannot move independently, relies on others for transportation, or uses powered devices for mobility in outdoor and community environments. Level V: Dependent on assistive technologies like wheelchairs, with severely restricted self-mobility.

#### EDACS/mini-EDACS level

EDACS is a system that classifies the eating functions of individuals with CP into five levels, with each level determined based on the eating efficiency and safety (25, 26). Level I: Eats and drinks safely and efficiently. Level II: Eats and drinks safely, though with some limitations in efficiency. Level III: Eats and drinks with some safety concerns; efficiency may also be limited. Level IV: Eats and drinks with major safety concerns. Level V: Unable to eat or drink safely; tube feeding may be considered for nutrition.

#### Anthropometric measurements

Anthropometric information including weight, length/height, and MUAC was obtained by two trained researchers following standard operating procedures to ensure consistency and accuracy (27). Weight was measured to the nearest 0.1 kg using a digital weight scale. Length/height and MUAC were measured to the nearest 0.1 cm with an anthropometer and anthropometric tape. Recumbent length was measured for participants under 24 months, while standing height was measured for those between 24 and 60 months. Estimated height was calculated using Stevenson’s equation [height (cm) = (tibial length × 3.26) + 30.8] (28) in situations where participants could not stand straight because of joint contractures or deformities. All participants were asked to remove heavy cloths and shoes prior to the measurement.

#### Classification of nutritional status

Nutritional status was classified by the weight-for-length/height Z score (WLZ/WHZ), weight-for-age Z score (WAZ), length/height-for-age Z score (LAZ/HAZ), and BMI-for-age Z score (BAZ). The z scores were calculated using WHO Anthro software (version 3.2.2). Malnutrition mentioned in this study includes undernutrition (underweight, stunting and wasting) and overnutrition (overweight and obesity) (29). According to WHO growth charts as well as the American Society for Parenteral and Enteral Nutrition (ASPEN) standards (13, 14, 30), mild wasting was defined as −2 < WLZ/WHZ/BAZ ≤ −1, moderate wasting was defined as −3 < WLZ/WHZ/BAZ ≤ −2, severe wasting was defined as WLZ/WHZ/BAZ ≤ −3; moderate stunting was defined as −3 < LAZ/HAZ ≤ −2, severe stunting was defined as LAZ/HAZ ≤ −3; moderate underweight was defined as −3 < WAZ ≤ −2, severe underweight was defined as WAZ ≤ −3; overweight was defined as 2<WLZ/WHZ//BAZ ≤ 3, whereas obesity was defined as WLZ/WHZ/BAZ > 3. Considering that a child may have multiple types of malnutrition categories, we used the most severe malnutrition level among the four Z Scores as the overall nutritional status. When there is a discrepancy between HAZ/LAZ and WHZ/WLZ or BAZ, we used the results from WHZ/WLZ or BAZ.

### Statistical analysis

Quantitative variables were presented as mean (standard deviations, SD) or median [interquartile ranges (IQR)], and categorical variables were presented as numbers or percentages. The sensitivity and specificity were calculated to investigate the diagnostic value of the MUAC cutoffs proposed by WHO. Kolmogorov–Smirnov test was used to assess the normality of the data. Spearman’s rank correlations were chosen to measure the direction and strength of the linear relationships between MUAC and four Z scores separately because the data of MUAC, WHZ, LAZ and BAZ did not follow a normal distribution. ROC curves and the area under the curve (AUC) were conducted to assess the sensitivity and specificity of MUAC cutoffs for different types and severities of malnutrition. The Youden Index was used to determine the optimal cutoff values for malnutrition. As selecting the MUAC cutoff with the highest sensitive would lead to a higher number of false positives, it is important to balance sensitivity with specificity by limiting false-positive rate to be below 33.33%. To assess the robustness of the optimal MUAC cutoffs, we performed a bootstrap resampling analysis with 1,000 iterations by using R software (version 4.5.1; R Foundation for Statistical Computing, Vienna, Austria). To evaluate whether the optimal MUAC cutoffs that we determined have the same predictive value for preschool children with CP of different severities, we additionally calculated the sensitivity and specificity of the optimal MUAC cutoff values among different GMFCS levels. All statistical analyses, except for the bootstrap resampling (1,000 iterations) analysis, were performed by using SPSS software (IBM SPSS Statistics for Windows, version 25.0; IBM Corp., Armonk, NY, United States).

## Results

A total of 937 children (606 boys and 331 girls) were included in the data analysis. The age of the sample population ranged from 12 to 60 months. The most common type of CP was spastic CP (85.17%). 52.51% of children were in GMFCS levels I and II and more than half were in the EDACS or Mini-EDACS level I. Nearly half of the children were born prematurely or had low birth weight. Sample characterization was showed in Table 1. Additionally, the height of 55 subjects was determined using estimation formulas. Table 2 showed that 53.68% participants were classified as malnourished, with the majority being categorized as undernourished (50.69%).

**Table 1:** The characteristics of the participants (*n* = 937).

Characteristics	Values, n (%) or Mean ± SD
Age (m), mean ± SD	32.5 ± 13.63
Sex, *n* (%)	
Male	606(64.67)
Female	331(35.33)
Gestational age	
<37 weeks	426(45.46)
≥37 weeks	511(54.54)
Birth weight	
<2.5 kg	413(44.08)
≥2.5 kg	524(55.92)
Primary motor type, *n* (%)	
Spastic	798(85.17)
Dyskinesia	81(8.64)
Ataxia	12(1.28)
Mixed	46(4.91)
GMFCS levels, *n* (%)	
I	287(30.63)
II	205(21.88)
III	172(18.36)
IV	155(16.54)
V	118(12.59)
EDACS/Mini-EDACS levels, *n* (%)	
I	554(59.12)
II	212(22.62)
III	123(13.13)
IV	38(4.06)
V	10(1.07)

Numbers are represented as n (%) or mean ± SD.

**Table 2:** The characteristics of the nutritional status (*n* = 937).

Nutritional status	*n* (%)		
	Mild	Moderate	Severe
Undernutrition	153(16.33)	211(22.52)	111(11.85)
Wasting (WLZ/WHZ)	247(26.36)	86(9.18)	38(4.05)
Underweight (WAZ)	/	146(15.58)	70(7.47)
Stunning (LAZ/HAZ)	/	165(17.61)	88(9.39)
Wasting (BAZ)	217(23.16)	74(7.90)	30(3.20)
Overweight	14(1.49)		
Obesity	14(1.49)		
Normal	434(46.32)		

Data are presented as n or percentage. WLZ, weight-for-length Z score; WHZ, weight-for-height Z score; WAZ, weight-for-age Z score; LAZ, length-for-age Z score; HAZ, height-for-age Z score; BAZ, BMI-for-age Z score.

### The validity of WHO-recommended MUAC cutoffs

According to WHO’s guidelines, in children aged 6–59 months whose MUAC was between 11.5 and 12.5 cm were classified as moderate undernutrition, and those under 11.5 cm were classified as severe undernutrition. The prevalence of different nutritional statuses among CP children aged 12–60 months was classified by Z scores and MUAC, as shown in Table 3. Notably, WHO-recommended MUAC cutoffs categorized fewer children as undernutrition compared to Z scores. WHO-recommended MUAC cutoffs categorized 37 (3.95%) children as undernutrition, while WLZ/WHZ, WAZ, LAZ/HAZ, and BAZ categorized 371 (39.59%), 216 (23.05%), 253 (27%), and 321 (34.26%) children as undernutrition, respectively.

**Table 3:** Prevalence of malnutrition identified by WHO-recommended MUAC cutoffs in children with CP (*n* = 937).

Variables	MUAC ≤ 11.5 cm	MUAC 11.5–12.5 cm	MUAC > 12.5 cm
WLZ/WHZ, *n* (%)			
Severe wasting	1(0.11)	7(0.75)	30(3.2)
Moderate wasting	2(0.21)	10(1.07)	74(7.9)
Mild wasting	1(0.11)	5(0.53)	241(25.72)
Normal	2(0.21)	5(0.53)	535(57.1)
Overweight	0	2(0.21)	13(1.39)
Obesity	0	2(0.21)	7(0.75)
WAZ, *n* (%)			
Severe underweight	4(0.43)	13(1.39)	53(5.66)
Moderate underweight	2(0.21)	7(0.75)	137(14.62)
Normal	0	11(1.17)	710(75.77)
LAZ/HAZ, *n* (%)			
Severe stunning	5(0.53)	14(1.49)	69(7.36)
Moderate stunning	1(0.11)	6(0.64)	158(16.86)
Normal	0	11(1.17)	673(71.82)
BAZ, *n* (%)			
Severe wasting	0	8(0.85)	22(2.35)
Moderate wasting	1(0.11)	6(0.64)	67(7.15)
Mild wasting	2(0.21)	6(0.64)	209(22.31)
Normal	3	7(0.75)	579(61.79)
Overweight	0	1(0.11)	12(1.28)
Obesity	0	3(0.32)	11(1.17)

Data are presented as n or percentage. MUAC, Mid-Upper Arm circumference; WLZ, weight-for-length Z score; WHZ, weight-for-height Z score; WAZ, weight-for-age Z score; LAZ, length-for-age Z score; HAZ, height-for-age Z score; BAZ, BMI-for-age Z score.

The currently used MUAC cutoffs recommended by WHO showed high specificity but low sensitivity compared to the Z score cutoffs. Positive predictive values (PPVs) of the MUAC cutoffs ranged from 0 to 83.33%, while negative predictive values (NPVs) maintained at a relatively higher level, ranging from 74.78–96.78% (Table 4).

**Table 4:** Sensitivity and specificity of WHO-recommended MUAC cutoffs in children with CP.

Variables	Sensitivity, %	Specificity, %	PPV, %	NPV, %
WLZ/WHZ				
Severe wasting	2.63	99.44	16.67	96.03
Moderate wasting	16.13	97.91	54.04	88.44
WAZ				
Severe underweight	5.71	99.77	66.67	92.91
Moderate underweight	12.04	98.47	70.27	78.89
LAZ/HAZ				
Severe stunning	5.68	99.88	83.33	91.08
Moderate stunning	10.28	98.39	70.27	74.78
BAZ				
Severe wasting	0	99.34	0	96.78
Moderate wasting	14.42	90.11	40.54	90.11

MUAC, Mid-Upper Arm circumference; WLZ, weight-for-length Z score; WHZ, weight-for-height Z score; WAZ, weight-for-age Z score; LAZ; length-for-age Z score; HAZ, height-for-age Z score; BAZ, BMI-for-age Z score; PPV, positive predictive value; NPV, negative predictive value.

### The correlation between MUAC and Z scores

**Figure 1:**
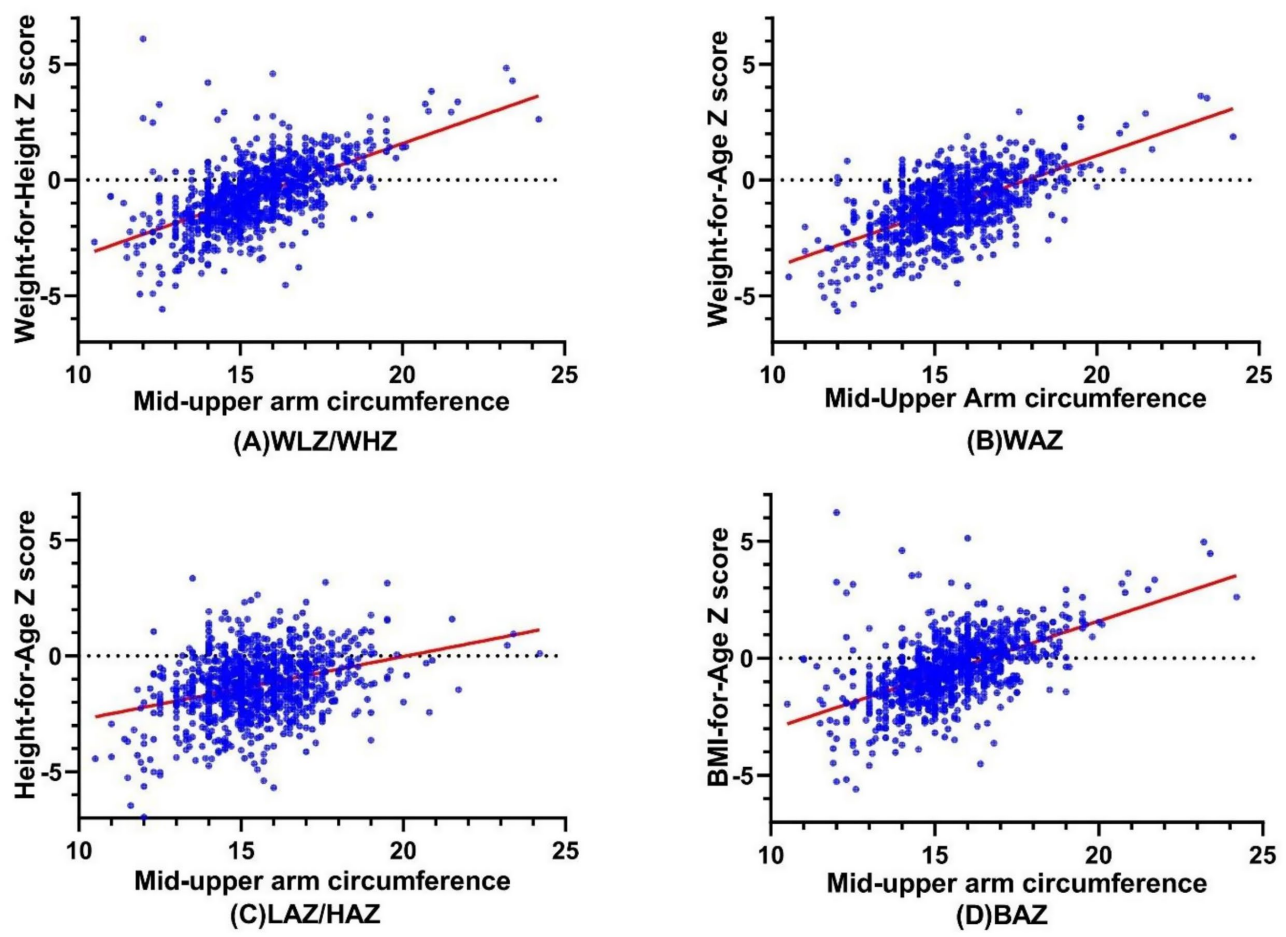
The Correlations between MUAC and **(A)** WLZ/WHZ, **(B)** WAZ, **(C)** LAZ/HAZ, and **(D)** BAZ. MUAC, Mid-Upper Arm circumference; WLZ, weight-for-length Z score; WHZ, weight-for-height Z score; WAZ, weight-for-age Z score; LAZ, length-for-age Z score; HAZ, height-for-age Z score; BAZ, BMI-for-age Z see.

Figure 1 shows the correlations between MUAC and WLZ/WHZ, WAZ, LAZ/HAZ, and BAZ, respectively. There was a statistically significant, strong correlation between MUAC and WLZ/WHZ (*r* = 0.606, *p* < 0.001), followed by WAZ (*r* = 0.557, *p* < 0.001) and BAZ (*r* = 0.575, *p* < 0.001). Meanwhile, there was a significant but weak correlation between MUAC and HAZ (*r* = 0.276, *p* < 0.001).

### The ability of MUAC to correctly identify malnutrition

**Figure 2:**
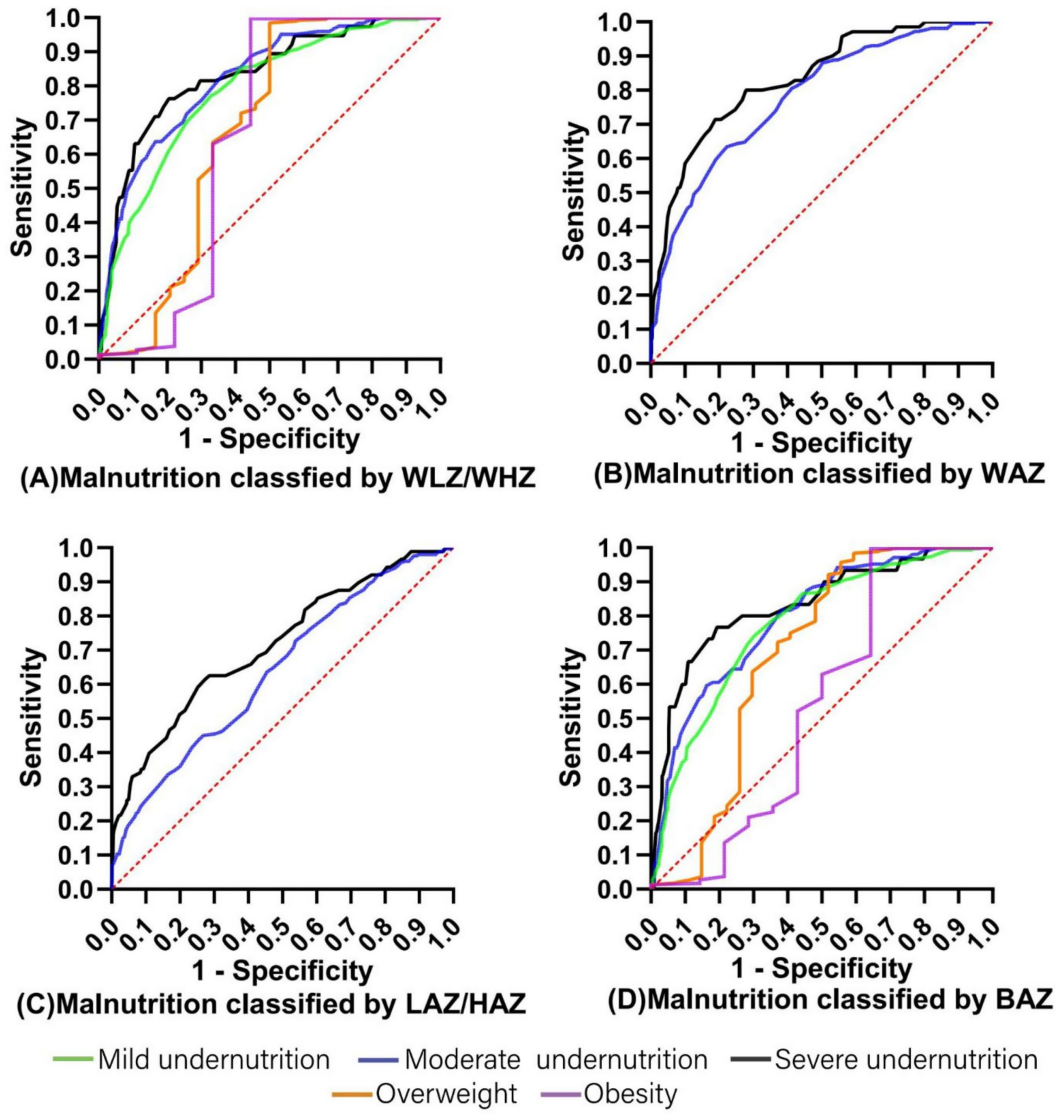
The ROC curves for MUAC prediction of malnutrition classified by **(A)** WLZ/WHZ, **(B)** WAZ, **(C)** LAZ/HAZ, and **(D)** BAZ. MUAC, Mid-Upper Arm circumference; WLZ, weight-for-length Z score; WHZ, weight-for-height Z score; WAZ, weight-for-age Z score; LAZ, length-for-age Z score; HAZ, height-for-age Z score; BAZ, BMI-for-age Z score.

ROC curves were conducted to assess the ability of MUAC to correctly predict malnutrition (mild undernutrition, moderate undernutrition, severe undernutrition, overweight, and obesity) (Figure 2). MUAC identified malnutrition with moderate accuracy (AUC ranged from 0.539 to 0.833).

Table 5 shows the optimal MUAC cutoffs for predicting malnutrition classified by WLZ/WHZ, WAZ, LAZ/HAZ, and BAZ in children with CP aged 12–60 months. In order to effectively identify CP children with or at risk of malnutrition, the optimal MUAC cutoffs for mild undernutrition, moderate undernutrition, severe undernutrition, overweight, and obesity were 15.35 cm (AUC 0.785), 15.05 cm (AUC 0.797), 14.35 cm (AUC 0.830), 17.55 cm (AUC 0.688), and 20.4 cm (AUC 0.651), respectively. Besides, the results of 1,000 bootstrap resampling were consistent with the above findings, indicating the robustness of the identified cutoffs (Table 6).

**Table 5:** Optimal MUAC cutoff for malnutrition classified by WHZ, WAZ, HAZ, BAZ.

Variables	Cutoff (cm)	Sensitivity, %	Specificity, %	False positive rates	AUC
WLZ/WHZ					
Severe wasting	14.35	79.4	76.3	0.237	0.830
Moderate wasting	14.55	77.6	67.7	0.323	0.822
Mild wasting	15.35	67.1	77.1	0.229	0.785
Overweight	18.95	50	98.5	0.015	0.669
Obesity	20.40	55.6	99.7	0.003	0.651
WAZ					
Severe underweight	14.35	81.1	71.4	0.286	0.833
Moderate underweight	15.05	65.5	71.8	0.282	0.778
LAZ/HAZ					
Severe stunning	15.15	57.1	68.2	0.318	0.715
Moderate stunning	15.75	46.2	72.7	0.273	0.639
BAZ					
Severe wasting	14.25	79.4	76.3	0.233	0.832
Moderate wasting	15.05	61.7	81.7	0.183	0.797
Mild wasting	15.15	69.8	74.1	0.259	0.777
Overweight	17.55	48.1	92.1	0.079	0.688
Obesity	20.40	35.7	99.7	0.003	0.539

MUAC, Mid-Upper Arm circumference; WLZ, weight-for-length Z score; WHZ, weight-for-height Z score; WAZ, weight-for-age Z score; LAZ, length-for-age Z score; HAZ, height-for-age Z score; BAZ, BMI-for-age Z score.

**Table 6:** Bootstrap-based validation (1,000 resamples) of MUAC diagnostic accuracy.

Variables	AUC (95%CI)	Sensitivity (95%CI)	Specificity (95%CI)
WLZ/WHZ			
Severe wasting	0.83 (0.76 ~ 0.89)	79.4 (50 ~ 89.5)	76.3 (60.5 ~ 89.5)
Moderate wasting	0.82 (0.78 ~ 0.86)	83.4 (72.7 ~ 88.1)	71.1 (50.0 ~ 84.2)
Mild wasting	0.79 (0.76 ~ 0.81)	67.1 (58.7 ~ 72.4)	77.1 (69.5 ~ 81.1)
Overweight	0.67 (0.52 ~ 0.82)	50 (25 ~ 66.7)	98.5 (53.8 ~ 100)
Obesity	0.65 (0.37 ~ 0.92)	55.6 (11.1 ~ 88.9)	62.9 (1.8 ~ 100)
WAZ			
Severe underweight	0.83 (0.78 ~ 0.88)	81.1 (60.7 ~ 87.2)	68.6 (58.5 ~ 80)
Moderate underweight	0.78 (0.75 ~ 0.81)	78.9 (63.4 ~ 83.1)	62 (51.8 ~ 68.5)
LAZ/HAZ			
Severe stunning	0.72 (0.66 ~ 0.77)	72.7 (51.5 ~ 80)	59.1 (47.7 ~ 70.5)
Moderate stunning	0.64 (0.60 ~ 0.68)	46.2 (34.5 ~ 50.6)	70.8 (62.1 ~ 77.9)
BAZ			
Severe wasting	0.83 (0.75 ~ 0.91)	80.7 (49.3 ~ 90.8)	79.7 (59.9 ~ 90)
Moderate wasting	0.80 (0.75 ~ 0.84)	61.7 (50.9 ~ 64.8)	81.7 (64.4 ~ 88.5)
Mild wasting	0.78 (0.75 ~ 0.81)	69.8 (61.9 ~ 73.9)	74.1 (59.5 ~ 79.1)
Overweight	0.69 (0.54 ~ 0.82)	48.1 (29.6 ~ 66.7)	92.1 (64.2 ~ 100)
Obesity	0.54 (0.33 ~ 0.72)	35.7 (0 ~ 57.1)	99.7 (24.8 ~ 100)

MUAC, Mid-Upper Arm circumference; WLZ, weight-for-length Z score; WHZ, weight-for-height Z score; WAZ, weight-for-age Z score; LAZ, length-for-age Z score; HAZ, height-for-age Z score; BAZ, BMI-for-age Z score.

Table 7 shows the predictive value of the optimal MUAC cutoffs for preschoolers with CP across GMFCS levels I–III and IV–V. It shows that the optimal MUAC cutoffs have good predictive value for both undernutrition and overnutrition in preschoolers with CP at GMFCS levels I to III. However, for children at GMFCS levels IV and V, the cutoff values show good predictive value only for undernutrition.

**Table 7:** Sensitivity and specificity of the optimal MUAC cutoffs in preschoolers with CP across different GMFCS levels.

GMFCS level	Nutritional status	Sensitivity, %	Specificity, %	PPV, %	NPV, %
I ~ V	Mild undernutrition	69.6	69.57	70.34	68.82
	Moderate undernutrition	59.57	65.58	47.77	75.42
	Severe undernutrition	56.64	81.8	29.91	93.22
	Overweight	48	92	14.12	98.47
	Obesity	38.46	99.57	55.56	99.14
I ~ III	Mild undernutrition	69.72	70.26	63.67	75.64
	Moderate undernutrition	54.91	66.60	36.68	80.74
	Severe undernutrition	42.21	84.35	14.04	96.00
	Overweight	64.71	90.73	15.49	98.99
	Obesity	62.50	99.54	62.50	99.54
IV&V	Mild undernutrition	69.43	66.25	83.23	47.32
	Moderate undernutrition	64.90	61.48	67.59	58.59
	Severe undernutrition	64.00	73.74	48.00	84.39
	Overweight	12.50	95.05	7.14	97.30
	Obesity	0	99.63	0	98.16

PPV, positive predictive value; NPV, negative predictive value.

## Discussion

MUAC is an extensively used tool to identify malnutrition, especially in resource-limited set-ups, and an essential indicator of associated mortality risk (31, 32). This study explored the use of MUAC and sought to identify the optimal cutoff values in outpatient preschoolers with CP aged 12–60 months.

Our results indicated that the MUAC cutoffs recommended by WHO seem inapplicable to children with CP. Compared to WLZ/WHZ, WAZ, LAZ/HAZ, and BAZ, the specificity and NPV of current MUAC cutoffs were excellent. However, the sensitivity and PPV were not good, indicating that the WHO-recommended MUAC cutoffs missed a substantial proportion of children with CP who suffered from undernutrition. WHO-recommended MUAC cutoffs identified fewer children with malnutrition because MUAC is just a single measurement of anthropometry, and the standard WHO cutoff values are not stratified by age or sex, whereas Z-scores are.

The correlations between MUAC with Z scores, MUAC and WLZ/WHZ, WAZ, and BAZ were more relevant than with LAZ/HAZ. This may be explained by MUAC measuring the sum of bone, muscle, and fat in the midpoint of the upper arm, which are more affected by weight than height. MUAC is closely related to weight, while segmental lengths, especially tibia length, are highly correlated with height (33, 34). Thus, MUAC may be unsuitable for screening malnutrition identified by HAZ, namely stunting.

The ESPGHAN working group highlighted five “red flags” for detecting undernutrition in children with neurological impairment: (1) physical signs of malnutrition (e.g., pressure-related skin lesions, poor peripheral circulation), (2) WAZ < −2, (3) triceps skinfold thickness (TSF) below the 10th percentile for age and sex, (4) mid-upper arm fat or muscle area (AMA) below the 10th percentile, and (5) faltering growth and/or failure to thrive (35). In outpatient settings, these “red flag” indicators may face practical challenges: anthropometric measures such as TSF and AMA require specialized equipment, trained personnel, and cooperative patients, which can be difficult in children with CP. In contrast, MUAC offers a simple, quick, and non-invasive alternative that is easy to perform in busy clinical and requires minimal training. Considering that the WHO-recommended MUAC cutoffs may underestimate the prevalence of undernutrition in children with CP, we seek to optimized the MUAC cutoffs to potentially improve the screening sensitivity and better identify those with malnutrition. Our previous study reported that malnutrition prevalence in children with CP was about 57.6%, comprising 50.8% undernutrition (14.77% mild, 23.89% moderate, 12.16% severe) and 6.8% overnutrition (6). The optimal MUAC cutoffs we determined achieved higher sensitivity than the WHO-recommended cutoffs. For mild undernutrition, the optimal MUAC cut-offs demonstrated balanced sensitivity and specificity (about 69%), with a PPV (70.3%) exceeding the pre-test probability. For overweight and obesity, low PPVs reflected the lower pre-test probability (6.8%), whereas NPVs >98% indicate high effectiveness in excluding excessive nutritional status. Overall, the optimal MUAC cut-offs appear to be a useful tool for screening malnutrition in preschoolers with CP in outpatient clinics.

With respect to the prediction of undernutrition classified by Z scores, the results of this study indicated that the optimal MUAC cutoffs might be 15.35, 15.05, and 14.35 cm for mild, moderate, and severe undernutrition, respectively. We found the optimal cutoffs slightly higher than those previously reported in typical developing Cambodian children by Fiorentino et al. (36). The difference can be explained by the fact that we calculated the optimal cutoff based on the data from all participants aged 12–60 months, while Fiorentino et al. split the participants into two age groups. Additionally, a considerable proportion of the children in this study were preterm, male, and low birth weight (37). Although the cutoffs in the two studies are slightly different, the present evidence suggests that an increase in the current cutoff values may improve the predictability of MUAC.

Additionally, the optimal MUAC cutoffs that we established showed low sensitivity for predicting overnutrition in preschoolers with CP at GMFCS levels IV and V. This may be due to the low proportion of overnourished children with CP at GMFCS levels IV and V in our sample. However, this situation is consistent with the distribution of malnutrition categories among children with CP, where the proportion of overnutrition is much lower than that of undernutrition (6). What is more, with the severity increases, children with CP are more likely to have additional comorbidities, which further hinder energy accumulation (6). Therefore, larger-sample studies are needed to validate the effectiveness of the optimal MUAC cutoffs that we identified or to establish more accurate MUAC cutoffs for predicting overnutrition in children with CP.

Being overweight or obese can negatively affect CP children’s rehabilitation management and social life. To the best of our knowledge, this is the first study that explored the ability of MUAC to identify overnutrition in children with CP (18). A small number of studies demonstrated that MUAC is a reliable tool for identifying over nutrition in typically developing children (19, 38). Talma et al. (39) enrolled children aged 2 to 18 in their study but only proposed the MUAC Z score cutoffs for overweight and obesity. Previous studies found the MUAC cutoff values for children over 5 years old with overnutrition (ranging from 19.05 to 29.8 cm), which are higher than the optimal cutoff value in this study (19, 40, 41). With the optimal cutoffs in our study, 9.37% of children were in overnutrition when only 2.13 and 2.45% were indicated by WHZ and BAZ. This can be explained by the WHO growth charts considering gender and age in months. Thus, more studies are needed to determine the optimal cutoffs that predict overnutrition in different age groups.

The main contribution of the study is that we evaluated the ability of WHO-recommended MUAC cutoffs for nutrition screening among children with CP and determined the most appropriate cutoffs for detecting undernutrition as well as overnutrition in outpatient CP children aged between 12 and 60 months. It is important to provide early, effective nutrition management and growth monitoring for children with CP since malnutrition is one of the common co-occurring conditions. Compared to standard questionnaires, such as Subjective Global Nutritional Assessment (42), MUAC, an objective measurement, is more convenient and reliable. The optimal MUAC cutoffs we found in this study could be used in busy clinics to identify children at risk of malnutrition.

The main limitation of this study is that we did not conduct a finer stratification for MUAC cutoffs since the cross-sectional design led to unequal allocation of participants in terms of sex, age, GMFCS levels, and EDACS/Mini-EDACS levels. And we did not explore whether birth conditions of children with CP affect the MUAC cutoffs, such as gestational age and birth weight. Besides, CP is often accompanied by epilepsy, intellectual disability, and feeding and swallowing disorders (43, 44) that may adversely affect an individual’s nutritional status. However, the influence of comorbidities on MUAC cutoffs was not the focus of this study. Moreover, MUAC may be unsuitable for screening overnutrition because there is no more accurate measurement to determine body composition, especially body fat, than the BMI-for-age Z score. Additionally, this study lacks external validation, which may limit the generalizability of the proposed MUAC cut-offs.

## Conclusion

MUAC is a potentially valuable tool for nutrition screening in children with CP aged 12 to 60 months. Further studies are needed to establish the MUAC cutoffs among children with CP over 5 years old and to verify whether there are differences related to the severity of motor and swallowing dysfunction.

## Data Availability

The raw data supporting the conclusions of this article will be made available by the authors, without undue reservation.
